# Supplementary material for: Pyrrolizidine Alkaloids Disturb Bile Acid Homeostasis in the Human Hepatoma Cell Line HepaRG
Source: Foods. 2021 Jan 14;10(1):161. doi: 10.3390/foods10010161 (PMC7828834; doi:10.3390/foods10010161)
Supplement: Supplementary file 1 [file foods-10-00161-s001.pdf]

- Supplemental material -

Pyrrolizidine alkaloids disturb bile acid homeostasis in the human hepatoma cell line HepaRG

Julia Waizenegger[1/2], Josephin Glück[1], Marcus Henricsson[3], Claudia Luckert[1], Albert Braeuning[1], Stefanie Hessel-Pras[1]

[1] German Federal Institute for Risk Assessment, Department of Food Safety, Max-Dohrn-Straße 8-10, 10589 Berlin, Germany

[2] German Nutrition Society, Godesberger Allee 18, 53175 Bonn, Germany

[3] Wallenberg Laboratory and Sahlgrenska Center for Cardiovascular and Metabolic Research, Institute of Medicine, University of Gothenburg, 413 45, Gothenborg, Sweden

Corresponding author:
Dr. Stefanie Hessel-Pras
ORC-ID: 0000-0002-6153-0035
German Federal Institute for Risk Assessment
Max-Dohrn-Str. 8-10
10589 Berlin, Germany
E-mail: stefanie.hessel-pras@bfr.bund.de
Tel.: +49-30-18412-25203

Table S1: Alterations of gene expressions of hepatobiliary transport proteins after 24 h PA treatment in HepaRG cells.

	Ctr		5 µM Em		35 µM Em		70 µM Em		5 µM Sc		35 µM Sc		70 µM Sc	
	**mean**	**SD**	**mean**	**SD**	**mean**	**SD**	**mean**	**SD**	**mean**	**SD**	**mean**	**SD**	**mean**	**SD**
ABCB1	1.1	0.0	-1.2	0.1	-2.7	0.8	-2.3	0.1	1.3	1.0	-2.0	0.8	-2.2	1.0
ABCB4	1.0	0.0	-1.8	0.2	-12.8	4.0	-24.5	0.1	-2.6	0.7	-22.5	4.0	-33.0	5.4
ABCB11	1.0	0.0	-6.1	0.4	-20.7	5.5	-16.6	0.6	-4.6	0.1	-18.7	4.8	-21.6	9.5
ABCC2	1.0	0.0	1.1	0.2	-2.3	0.2	-3.3	0.4	-1.1	0.1	-3.5	0.2	-5.0	0.5
ABCC3	1.0	0.0	-1.3	0.2	-2.6	1.0	-4.0	1.1	-1.4	0.2	-3.2	0.9	-3.6	0.6
ABCC6	1.0	0.0	-1.5	0.2	-10.9	1.8	-19.3	1.7	-1.9	0.3	-23.2	5.8	-37.7	8.3
SLC10A1	1.0	0.0	-2.6	1.4	-79.6	4.1	-120.5	85.7	-10.1	6.8	-165.1	65.6	-179.5	117.0
SLC22A7	1.0	0.0	-2.5	1.3	-224.3	154.3	-689.9	270.3	-3.5	1.7	-542.9	169.3	-942.1	331.4
SLC22A9	1.0	0.0	-1.7	0.2	-31.7	5.0	-141.1	12.0	-3.3	0.6	-208.2	38.7	-1338.9	731.1
SLC51A	1.1	0.0	-3.9	2.2	-452.0	336.1	-265.2	23.8	-7.7	5.0	-567.7	254.3	-1575.4	206.2
SLC51B	1.2	0.2	-1.4	0.1	-3.6	1.6	-3.3	1.4	1.1	0.4	-2.8	0.8	-3.2	0.2
SLCO1B1	1.0	0.0	-1.1	0.2	-12.8	7.9	-43.1	4.5	-1.4	0.3	-32.5	4.0	-85.7	15.8
SLCO1B3	1.1	0.0	-1.8	0.7	1.6	0.2	-1.5	0.9	-1.8	0.6	1.4	0.4	2.5	2.2
SLCO2B1	1.0	0.0	-2.6	1.5	-52.6	20.2	-159.3	17.3	-4.1	1.5	-128.6	3.0	-359.7	41.2

	Ctr		5 µM Hn		35 µM Hn		70 µM Hn		5 µM Sk		35 µM Sk		70 µM Sk	
	**mean**	**SD**	**mean**	**SD**	**mean**	**SD**	**mean**	**SD**	**mean**	**SD**	**mean**	**SD**	**mean**	**SD**
ABCB1	1.1	0.0	-1.1	0.4	1.4	1.2	-1.4	0.2	1.1	0.6	1.1	0.7	1.0	0.6
ABCB4	1.0	0.0	-1.2	0.2	-2.9	0.7	-5.2	0.7	-1.3	0.2	-6.8	2.8	-12.2	3.7
ABCB11	1.0	0.0	-1.6	0.4	-12.4	3.3	-16.5	5.7	-2.0	0.3	-20.0	5.1	-20.4	2.9
ABCC2	1.0	0.0	1.1	0.1	-1.4	0.3	-2.4	0.9	1.3	0.3	-1.6	0.2	-2.2	0.0
ABCC3	1.0	0.0	-1.2	0.2	-1.7	0.5	-1.8	0.5	-1.0	0.1	-1.9	0.4	-2.3	0.5
ABCC6	1.0	0.0	-1.1	0.2	-3.0	0.9	-7.8	1.6	-1.2	0.3	-6.1	1.4	-11.5	2.7
SLC10A1	1.0	0.0	1.0	0.5	-8.9	3.9	-20.7	16.4	-1.5	0.7	-21.1	20.1	-55.0	51.8
SLC22A7	1.0	0.0	1.0	0.2	-7.9	5.7	-38.3	33.2	-1.2	0.2	-16.2	4.6	-53.8	26.3
SLC22A9	1.0	0.0	-1.2	0.2	-3.4	1.3	-7.9	0.7	-1.4	0.2	-12.4	1.6	-27.9	3.4
SLC51A	1.1	0.0	-1.3	0.6	-6.0	3.0	-41.6	11.0	-2.5	2.3	-160.4	37.7	-232.2	75.6
SLC51B	1.2	0.2	1.0	0.1	-1.8	0.5	-1.9	0.4	1.1	0.4	-2.0	0.2	-3.2	1.9
SLCO1B1	1.0	0.0	-1.1	0.2	-2.4	0.8	-5.7	2.9	-1.1	0.1	-5.3	1.3	-14.7	2.0
SLCO1B3	1.1	0.0	-1.6	0.2	-2.3	0.7	1.3	0.3	1.1	0.6	-1.7	0.4	1.1	0.7
SLCO2B1	1.0	0.0	-1.3	0.5	-6.7	4.0	-22.6	7.9	-1.9	0.6	-28.1	10.6	-80.4	14.6

Complete list of gene expressions of hepatobiliary transporters analyzed by qRT-PCR, which were regulated by the PA echimidine (Em), heliotrine (Hn), senecionine (Sc) and senkirkine (Sk) after 24 h treatment in HepaRG cells. Gene expressions were determined by the $\Delta\Delta C_t$ method and normalized to β-actin and the solvent control (Ctr). Data are summarized as mean ± standard deviation (SD) of three independent experiments with three replicates each.

Table S2: Alterations of gene expressions of hepatobiliary transport proteins after 14 days PA treatment in HepaRG cells.

	**Ctr**		**5 µM Em**		**35 µM Em**		**70 µM Em**		**5 µM Sc**		**35 µM Sc**		**70 µM Sc**	
	**mean**	**SD**	**mean**	**SD**	**mean**	**SD**	**mean**	**SD**	**mean**	**SD**	**mean**	**SD**	**mean**	**SD**
ABCB1	1.0	0.0	1.2	0.6	-1.5	0.1	-2.0	0.1	-1.2	0.3	-2.4	0.8	-2.3	0.7
ABCB4	1.0	0.0	-1.4	0.1	-5.7	2.6	-9.5	4.7	-1.9	0.6	-5.4	0.8	-9.9	1.4
ABCB11	1.0	0.0	-3.2	2.1	-43.1	4.3	-49.5	10.0	-4.0	2.2	-53.2	5.8	-107.3	85.6
ABCC2	1.0	0.1	1.1	0.2	-2.6	0.6	-3.2	0.8	-1.1	0.2	-3.3	0.9	-3.6	0.4
ABCC3	1.0	0.0	-1.0	0.1	-1.3	0.2	-1.2	0.1	-1.2	0.2	-1.4	0.3	-1.3	0.1
ABCC6	1.0	0.0	-1.2	0.1	-5.5	1.8	-8.0	3.4	-1.9	0.8	-9.4	3.4	-7.5	0.3
SLC10A1	1.0	0.0	-1.3	0.2	-4.7	1.5	-5.9	0.9	-1.4	0.2	-7.0	2.2	-10.2	3.6
SLC22A7	1.0	0.0	-2.0	0.8	-40.8	13.6	-135.7	76.3	-2.6	1.0	-115.6	42.0	-225.1	83.7
SLC22A9	1.0	0.0	-1.2	0.1	-5.5	2.0	-11.4	1.1	-1.2	0.4	-7.3	2.7	-14.5	0.4
SLC51A	1.0	0.2	-7.9	0.6	-399.0	67.6	-1002.5	106.1	-5.7	1.3	-197.0	48.4	-365.9	178.1
SLC51B	1.0	0.0	2.2	1.9	-5.2	2.8	-2.1	1.0	1.1	0.8	-1.8	0.1	-3.6	1.5
SLCO1B1	1.0	0.0	-1.4	0.3	-7.2	2.9	-17.1	3.7	-1.4	0.3	-13.6	1.5	-25.0	5.7
SLCO1B3	1.0	0.9	-1.6	0.4	1.6	0.4	1.6	0.4	1.1	0.6	2.4	1.9	4.1	3.8
SLCO2B1	1.1	0.1	-1.2	0.3	-12.5	11.4	-27.1	14.6	-1.3	0.3	-15.4	9.0	-28.9	12.8
	**Ctr**		**5 µM Hn**		**35 µM Hn**		**70 µM Hn**		**5 µM Sk**		**35 µM Sk**		**70 µM Sk**	
	**mean**	**SD**	**mean**	**SD**	**mean**	**SD**	**mean**	**SD**	**mean**	**SD**	**mean**	**SD**	**mean**	**SD**
ABCB1	1.0	0.0	-1.1	0.1	-1.3	0.6	-1.4	0.3	-1.8	1.5	-1.4	0.1	-1.4	0.5
ABCB4	1.0	0.0	-1.6	0.3	-3.3	1.1	-3.7	0.2	-1.6	0.6	-3.9	2.8	-4.5	2.3
ABCB11	1.0	0.0	-2.9	1.4	-21.9	4.1	-29.6	2.3	-2.4	1.3	-37.1	23.0	-54.6	26.0
ABCC2	1.0	0.1	1.0	0.3	-1.8	0.5	-2.2	0.6	1.0	0.3	-1.6	0.7	-2.0	0.8
ABCC3	1.0	0.0	-1.1	0.1	-1.3	0.3	-1.4	0.1	-1.1	0.1	-1.3	0.2	-1.2	0.2
ABCC6	1.0	0.0	-1.4	0.2	-4.3	1.4	-6.3	1.9	-1.7	0.8	-2.6	0.4	-6.9	3.3
SLC10A1	1.0	0.0	-1.0	0.1	-2.6	0.3	-4.6	1.6	-1.2	0.2	-2.2	0.5	-3.8	0.8
SLC22A7	1.0	0.0	-2.1	0.4	-13.7	4.7	-36.7	17.1	-1.9	0.7	-14.7	4.8	-20.5	4.4
SLC22A9	1.0	0.0	-1.2	0.1	-4.0	1.9	-4.0	1.8	-1.1	0.3	-2.5	0.8	-3.7	1.1
SLC51A	1.0	0.2	-4.1	1.3	-16.7	2.2	-30.2	9.7	-3.4	1.6	-39.3	15.4	-137.0	20.3
SLC51B	1.0	0.0	-1.4	0.5	-2.4	0.3	-1.6	0.2	1.1	0.8	-1.5	0.7	-2.8	1.3
SLCO1B1	1.0	0.0	-1.5	0.4	-3.4	0.9	-5.3	1.7	-1.1	0.2	-3.0	0.5	-4.7	0.9
SLCO1B3	1.0	0.9	1.2	1.1	2.3	1.1	4.1	3.0	1.9	1.2	1.4	0.9	2.3	1.1
SLCO2B1	1.1	0.1	-1.3	0.3	-2.6	1.1	-4.5	1.0	1.2	0.2	-2.5	0.2	-5.3	1.7

Complete list of gene expressions of hepatobiliary transporters analyzed by qRT-PCR, which were regulated by the PA echimidine (Em), heliotrine (Hn), senecionine (Sc) and senkirkine (Sk) after 14 days treatment in HepaRG cells. Gene expressions were determined by the $\Delta\Delta C_t$ method and normalized to β-actin and the solvent control (Ctr). Data are summarized as mean ± standard deviation (SD) of three independent experiments with three replicates each.

Table S3: Alterations of gene expressions of enzymes involved in bile acid/cholesterol metabolism after 24 h PA treatment in HepaRG cells.

	Ctr		5 µM Em		35 µM Em		70 µM Em		5 µM Sc		35 µM Sc		70 µM Sc	
	**mean**	**SD**	**mean**	**SD**	**mean**	**SD**	**mean**	**SD**	**mean**	**SD**	**mean**	**SD**	**mean**	**SD**
BAAT	1.0	0.0	-1.8	0.8	-30.4	6.5	-37.8	15.5	-2.5	0.8	-45.2	12.6	-58.7	19.8
CYP3A4	1.0	0.0	1.1	0.7	-90.2	42.6	-459.4	210.9	-2.1	1.1	-494.9	78.3	-1396.7	294.0
CYP7A1	1.0	0.0	-4.8	1.3	-953.0	387.0	-1914.4	382.4	-6.3	4.7	-2629.3	1203.9	-2686.3	75.9
CYP8B1	1.0	0.0	-1.7	0.1	-74.4	4.8	-269.7	7.0	-2.8	0.2	-605.4	94.3	-2559.5	73.6
CYP27A1	1.0	0.1	-1.2	0.3	-2.3	0.8	-2.8	0.6	-1.6	0.4	-2.5	1.1	-3.3	1.1
CYP39A1	1.1	0.1	-2.2	0.7	-5.3	0.3	-4.7	1.4	-3.4	0.3	-4.0	0.5	-6.4	0.4
SULT2A1	1.0	0.0	-2.1	0.8	-117.0	105.9	-212.6	64.4	-3.0	1.4	-340.8	119.5	-849.9	208.5
UGT2B4	1.1	0.1	1.2	0.3	-12.9	3.1	-31.8	2.9	-1.3	0.4	-48.1	2.9	-129.0	8.7
	**Ctr**		**5 µM Hn**		**35 µM Hn**		**70 µM Hn**		**5 µM Sk**		**35 µM Sk**		**70 µM Sk**	
	**mean**	**SD**	**mean**	**SD**	**mean**	**SD**	**mean**	**SD**	**mean**	**SD**	**mean**	**SD**	**mean**	**SD**
BAAT	1.0	0.0	-1.2	0.2	-3.5	1.8	-5.6	1.8	-1.3	0.3	-6.0	0.5	-13.2	2.1
CYP3A4	1.0	0.0	1.3	0.8	-4.5	0.6	-7.2	4.4	1.1	0.6	-14.7	5.2	-109.7	20.9
CYP7A1	1.0	0.0	1.3	0.8	-60.0	41.7	-585.5	332.2	-1.7	0.8	-360.6	312.7	-1353.5	55.5
CYP8B1	1.0	0.0	1.3	0.4	-3.7	0.3	-12.6	2.0	-1.2	0.5	-23.7	7.2	-35.3	14.6
CYP27A1	1.0	0.1	-1.0	0.0	-1.8	0.3	-2.3	0.5	-1.3	0.1	-2.7	0.1	-2.7	0.2
CYP39A1	1.1	0.1	-1.3	0.1	-3.2	0.8	-4.3	0.5	-1.5	0.4	-4.1	0.0	-5.3	0.9
SULT2A1	1.0	0.0	-1.2	0.2	-4.6	1.5	-14.5	3.4	-1.5	0.4	-17.1	6.1	-75.5	47.0
UGT2B4	1.1	0.1	2.2	0.7	-1.2	0.4	-3.8	2.5	1.4	0.2	-3.4	1.4	-18.6	2.9

Incomplete list of gene expressions of enzymes involved in bile acid/cholesterol metabolism analyzed by qRT-PCR, which were regulated by the PA echimidine (Em), heliotrine (Hn), senecionine (Sc) and senkirkine (Sk) after 24 h treatment in HepaRG cells. Gene expressions were determined by the $\Delta\Delta C_t$ method and normalized to β-actin and the solvent control (Ctr). Data are summarized as mean ± standard deviation (SD) of three independent experiments with three replicates each.

Table S4: Alterations of gene expressions of enzymes involved in bile acid/cholesterol metabolism after 14 days PA treatment in HepaRG cells.

	**Ctr**		**5 µM Em**		**35 µM Em**		**70 µM Em**		**5 µM Sc**		**35 µM Sc**		**70 µM Sc**	
	**mean**	**SD**	**mean**	**SD**	**mean**	**SD**	**mean**	**SD**	**mean**	**SD**	**mean**	**SD**	**mean**	**SD**
BAAT	1.0	0.0	-1.8	0.8	-30.4	6.5	-37.8	15.5	-2.5	0.8	-45.2	12.6	-58.7	19.8
CYP3A4	1.0	0.0	1.1	0.7	-90.2	42.6	-459.4	210.9	-2.1	1.1	-494.9	78.3	-1396.7	294.0
CYP7A1	1.0	0.0	-4.8	1.3	-953.0	387.0	-1914.4	382.4	-6.3	4.7	-2629.3	1203.9	-2686.3	75.9
CYP8B1	1.0	0.0	-1.7	0.1	-74.4	4.8	-269.7	7.0	-2.8	0.2	-605.4	94.3	-2559.5	73.6
CYP27A1	1.0	0.1	-1.2	0.3	-2.3	0.8	-2.8	0.6	-1.6	0.4	-2.5	1.1	-3.3	1.1
CYP39A1	1.1	0.1	-2.2	0.7	-5.3	0.3	-4.7	1.4	-3.4	0.3	-4.0	0.5	-6.4	0.4
SULT2A1	1.0	0.0	-2.1	0.8	-117.0	105.9	-212.6	64.4	-3.0	1.4	-340.8	119.5	-849.9	208.5
UGT2B4	1.1	0.1	1.2	0.3	-12.9	3.1	-31.8	2.9	-1.3	0.4	-48.1	2.9	-129.0	8.7

	**Ctr**		**5 µM Hn**		**35 µM Hn**		**70 µM Hn**		**5 µM Sk**		**35 µM Sk**		**70 µM Sk**	
	**mean**	**SD**	**mean**	**SD**	**mean**	**SD**	**mean**	**SD**	**mean**	**SD**	**mean**	**SD**	**mean**	**SD**
BAAT	1.0	0.0	-1.2	0.2	-3.5	1.8	-5.6	1.8	-1.3	0.3	-6.0	0.5	-13.2	2.1
CYP3A4	1.0	0.0	1.3	0.8	-4.5	0.6	-7.2	4.4	1.1	0.6	-14.7	5.2	-109.7	20.9
CYP7A1	1.0	0.0	1.3	0.8	-60.0	41.7	-585.5	332.2	-1.7	0.8	-360.6	312.7	-1353.5	55.5
CYP8B1	1.0	0.0	1.3	0.4	-3.7	0.3	-12.6	2.0	-1.2	0.5	-23.7	7.2	-35.3	14.6
CYP27A1	1.0	0.1	-1.0	0.0	-1.8	0.3	-2.3	0.5	-1.3	0.1	-2.7	0.1	-2.7	0.2
CYP39A1	1.1	0.1	-1.3	0.1	-3.2	0.8	-4.3	0.5	-1.5	0.4	-4.1	0.0	-5.3	0.9
SULT2A1	1.0	0.0	-1.2	0.2	-4.6	1.5	-14.5	3.4	-1.5	0.4	-17.1	6.1	-75.5	47.0
UGT2B4	1.1	0.1	2.2	0.7	-1.2	0.4	-3.8	2.5	1.4	0.2	-3.4	1.4	-18.6	2.9

Incomplete list of gene expressions of enzymes involved in bile acid/cholesterol metabolism analyzed by qRT-PCR, which were regulated by the PA echimidine (Em), heliotrine (Hn), senecionine (Sc) and senkirkine (Sk) after 14 days treatment in HepaRG cells. Gene expressions were determined by the $\Delta\Delta C_t$ method and normalized to β-actin and the solvent control (Ctr). Data are summarized as mean ± standard deviation (SD) of three independent experiments with three replicates each.

Table S5: Alterations of gene expressions of transcription regulators involved in bile acid/cholesterol metabolism after 24 h PA treatment in HepaRG cells.

	Ctr		5 µM Em		35 µM Em		70 µM Em		5 µM Sc		35 µM Sc		70 µM Sc	
	mean	SD	mean	SD	mean	SD	mean	SD	mean	SD	mean	SD	mean	SD
CAR	1.0	0.0	-3.4	1.7	-403.8	204.6	-1228.4	298.9	-6.7	3.0	-927.8	301.1	-1655.4	127.4
Erα	1.0	0.1	-3.8	1.8	-5.7	1.3	-4.5	1.8	-3.5	0.3	-4.5	0.8	-3.9	1.0
FXR	1.0	0.0	-1.3	0.1	-4.7	1.1	-9.1	2.3	-1.5	0.2	-7.9	0.6	-25.0	1.0
HNF1α	1.0	0.0	-1.1	0.0	-3.4	0.7	-5.6	1.3	-1.5	0.3	-5.1	0.4	-6.6	1.3
HNF4α	1.0	0.0	-1.4	0.2	-9.3	1.3	-15.3	0.2	-1.6	0.1	-14.9	2.4	-22.5	4.8
INSIG2	1.0	0.0	-3.1	0.6	-7.6	2.0	-7.8	1.0	-3.6	1.1	-6.6	2.4	-9.7	3.5
PPARα	1.1	0.1	-2.0	0.7	-7.5	1.7	-11.5	1.2	-2.6	0.7	-10.8	2.2	-12.5	1.2
PXR	1.0	0.0	-1.3	0.4	-14.4	1.1	-30.1	10.5	-2.1	0.4	-23.5	1.8	-46.5	26.8
SHP	0.9	0.2	-3.0	2.5	-6.2	1.6	-12.5	1.8	-2.3	0.3	-16.8	1.8	-20.0	3.6
SREBF1	1.0	0.0	-2.3	0.4	-6.2	1.6	-7.8	1.4	-3.9	1.9	-8.6	0.7	-8.9	0.7

	Ctr		5 µM Hn		35 µM Hn		70 µM Hn		5 µM Sk		35 µM Sk		70 µM Sk	
	mean	SD	mean	SD	mean	SD	mean	SD	mean	SD	mean	SD	mean	SD
CAR	1.0	0.0	-1.2	0.1	-5.1	3.2	-18.9	11.5	-1.7	0.3	-56.2	34.0	-217.4	113.8
Erα	1.0	0.1	2.4	0.5	-3.7	0.8	-5.0	0.7	-3.9	1.6	-6.6	1.3	-7.0	0.5
FXR	1.0	0.0	-1.1	0.2	-2.2	0.6	-4.0	1.0	-1.3	0.3	-4.1	0.9	-6.3	2.3
HNF1α	1.0	0.0	-1.2	0.3	-2.2	0.4	-2.9	0.2	-1.2	0.2	-2.5	0.6	-4.0	0.9
HNF4α	1.0	0.0	-1.3	0.2	-3.3	0.4	-5.5	0.8	-1.3	0.0	-5.3	0.6	-8.8	1.4
INSIG2	1.0	0.0	-1.5	0.3	-5.5	1.2	-8.6	2.1	-2.5	0.8	-9.1	1.7	-8.6	3.3
PPARα	1.1	0.1	-1.4	0.3	-3.6	0.4	-6.6	1.3	-1.5	0.2	-5.3	2.3	-7.7	2.8
PXR	1.0	0.0	1.0	0.1	-1.5	0.1	-3.6	1.4	-1.3	0.2	-5.7	0.2	-10.2	5.1
SHP	0.9	0.2	-1.7	1.3	-1.8	1.5	-2.1	1.1	-2.3	1.2	-4.3	2.3	-6.9	2.6
SREBF1	1.0	0.0	-1.4	0.3	-5.1	1.3	-6.3	0.6	-1.4	0.2	-6.9	2.9	-8.1	2.0

Complete list of gene expressions of transcription regulators involved in bile acid/cholesterol metabolism analyzed by qRT-PCR, which were regulated by the PA echimidine (Em), heliotrine (Hn), senecionine (Sc) and senkirkine (Sk) after 24 h treatment in HepaRG cells. Gene expressions were determined by the $\Delta\Delta C_t$ method and normalized to β-actin and the solvent control (Ctr). Data are summarized as mean ± standard deviation (SD) of three independent experiments with three replicates each.

Table S6: Alterations of gene expressions of transcription regulators involved in bile acid/cholesterol metabolism after 14 days PA treatment in HepaRG cells.

	Ctr		5 µM Em		35 µM Em		70 µM Em		5 µM Sc		35 µM Sc		70 µM Sc	
	mean	SD	mean	SD	mean	SD	mean	SD	mean	SD	mean	SD	mean	SD
CAR	1.0	0.0	1.4	1.2	-58.5	19.0	-125.2	32.1	1.2	0.6	-54.4	0,2	-144.0	3.1
Erα	1.0	0.0	-2.4	0.1	-2.3	1.2	-1.4	0.3	-1.5	0.4	-1.4	0.2	-1.3	0.6
FXR	1.0	0.0	1.0	0.1	-1.6	0.2	-1.9	0.1	1.0	0.3	-2.2	0.5	-3.7	0.9
HNF1α	1.0	0.1	-1.1	0.1	-2.2	1.1	-2.9	0.4	-1.3	0.6	-2.3	0.7	-3.3	1.0
HNF4α	1.0	0.0	-1.2	0.0	-3.7	1.1	-6.0	1.2	-1.4	0.4	-5.0	0.5	-8.3	0.7
INSIG2	1.0	0.0	-1.3	0.4	-7.9	3.0	-7.0	0.9	-1.2	0.5	-7.1	0.1	-7.3	2.2
PPARα	1.0	0.1	1.1	0.3	-1.7	0.3	-2.1	0.3	1.3	0.9	-2.1	0.4	-1.5	0.7
PXR	1.1	0.1	-1.2	0.2	-5.7	2.1	-15.4	0.9	-1.3	0.2	-11.2	0.8	-29.0	2.2
SHP	1.0	0.0	1.0	0.1	-6.4	3.8	-38.0	5.4	-1.2	0.3	-23.8	5.2	-200.0	122.9
SREBF1	1.0	0.0	-1.6	0.3	-20.3	7.0	-21.6	0.5	-2.4	0.7	-13.4	4.2	-16.4	7.9
	**Ctr**		**5 µM Hn**		**35 µM Hn**		**70 µM Hn**		**5 µM Sk**		**35 µM Sk**		**70 µM Sk**	
	**mean**	**SD**	**mean**	**SD**	**mean**	**SD**	**mean**	**SD**	**mean**	**SD**	**mean**	**SD**	**mean**	**SD**
CAR	1.0	0.0	1.2	0.8	-3.0	1.7	-5.5	0.1	1.5	0.8	-3.2	2.1	-5.2	2.6
Erα	1.0	0.0	-1.9	1.2	-1.3	0.7	-1.8	1.1	-1.6	0.5	-1.5	0.3	-1.9	0.8
FXR	1.0	0.0	1.0	0.1	-1.2	0.3	-1.3	0.4	1.2	0.2	-1.1	0.2	-1.4	0.2
HNF1α	1.0	0.1	-1.3	0.4	-1.6	0.9	-2.2	1.4	1.1	0.5	-1.8	1.0	-3.3	3.6
HNF4α	1.0	0.0	-1.1	0.1	-2.7	0.5	-3.6	0.3	-1.4	0.4	-2.3	1.0	-3.4	0.8
INSIG2	1.0	0.0	-1.4	0.4	-2.8	0.7	-3.9	0.4	-1.1	0.2	-3.0	1.1	-4.2	1.6
PPARα	1.0	0.1	-1.2	0.1	1.0	0.6	-2.0	0.1	-1.3	0.1	-1.7	0.6	-1.9	1.4
PXR	1.1	0.1	-1.3	0.2	-2.5	0.2	-3.7	0.7	1.0	0.2	-2.4	0.2	-4.3	2.0
SHP	1.0	0.0	-1.1	0.1	-2.9	1.1	-8.5	0.8	-1.1	0.2	-2.6	1.7	-4.1	2.0
SREBF1	1.0	0.0	-1.7	0.1	-5.9	1.3	-14.9	3.2	-1.8	0.5	-7.6	3.4	-8.0	2.5

Complete list of gene expressions of transcription regulators involved in bile acid/cholesterol metabolism analyzed by qRT-PCR, which were regulated by the PA echimidine (Em), heliotrine (Hn), senecionine (Sc) and senkirkine (Sk) after 14 days treatment in HepaRG cells. Gene expressions were determined by the $\Delta\Delta C_t$ method and normalized to β-actin and the solvent control (Ctr). Data are summarized as mean ± standard deviation (SD) of three independent experiments with three replicates each.

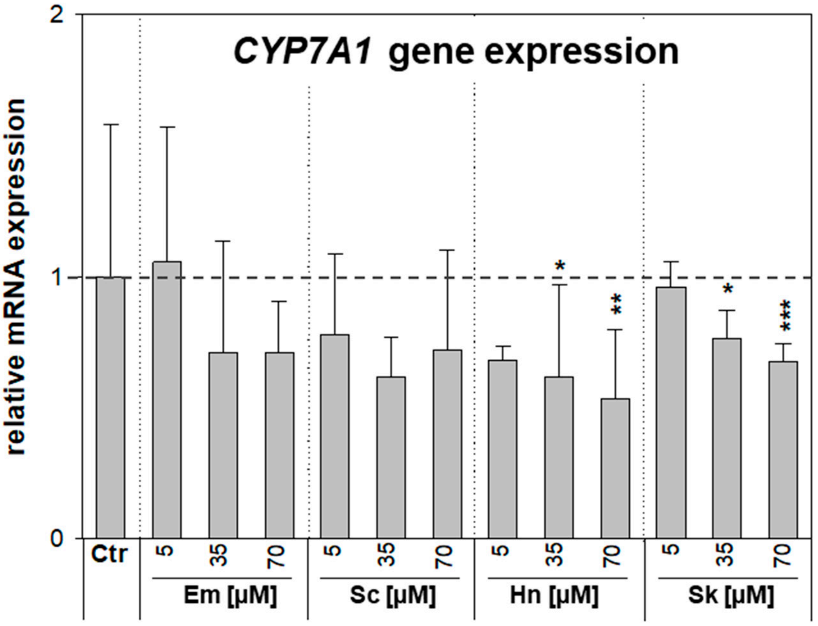

Figure S1: Influence of PA on CYP7A1 gene expression in HepG2 cells. For gene expression analysis cells were treated as indicated in the figure (Ctr; 2.5% ACN) for 24 h. Gene expression was analyzed as described in materials and methods. Gene expression of the target gene was normalized to ACTB and to solvent control to calculate relative expression ($\Delta\Delta C_t$ method). Shown are means ± standard deviations of three biological replicates. Statistical differences were calculated using one-way ANOVA followed by Dunnett's test: *$p<0.05$, **$p<0.005$, ***$p<0.001$. Em, echimidine; Sc, senecionine; Hn, heliotrine; Sk, senkirkine; PMA, phorbol-12-myristate-13-acetate

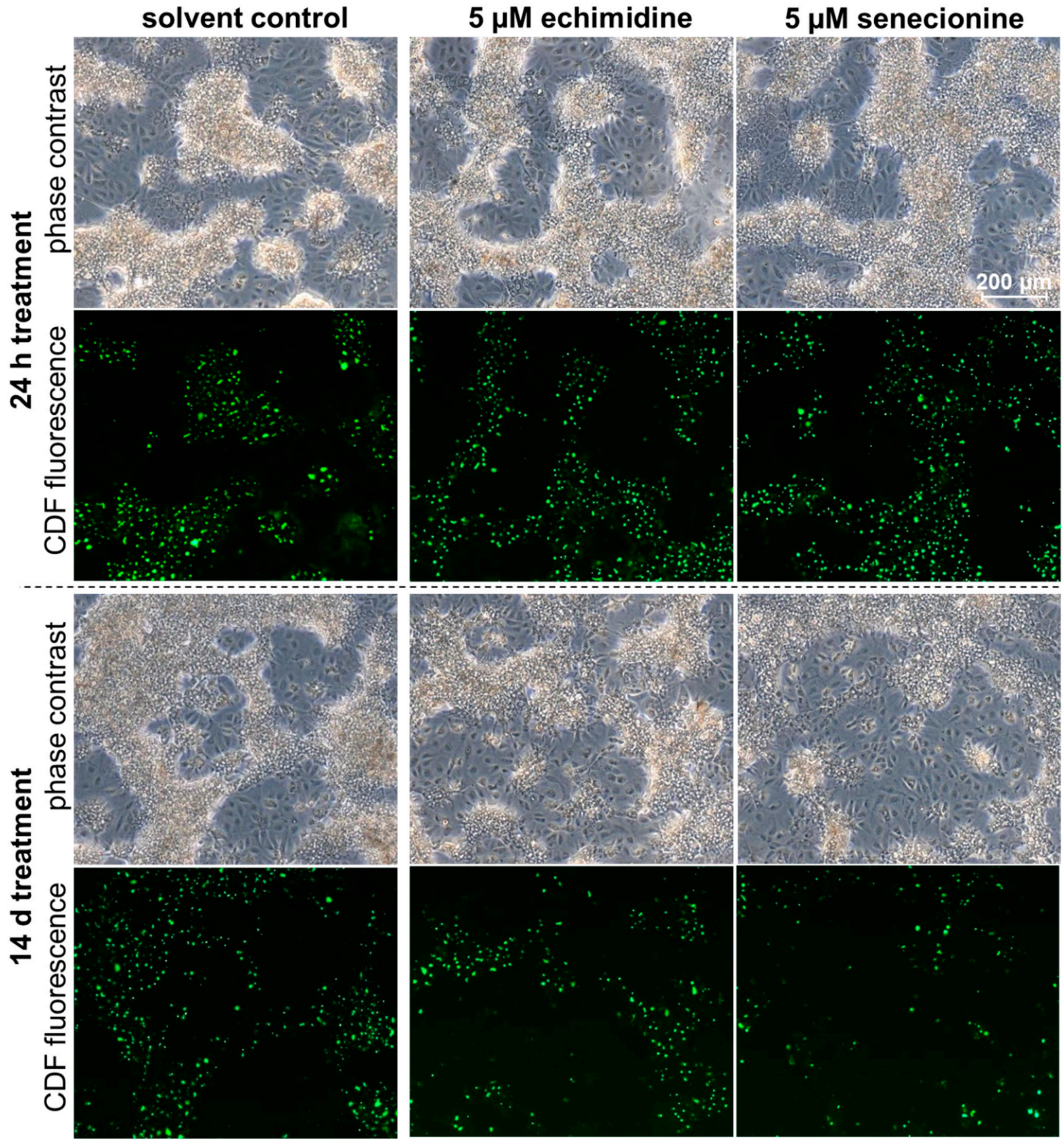

Figure S2: PA-dependent disturbance of ABCC-2 driven efflux in HepaRG cells: Differentiated HepaRG cells were incubated for 24 h or 14 days with 5 µM echimidine or senecionine as well as with the solvent (1.7 % DMSO and 0.35 % ACN). To localize the bile canaliculi, the cells were incubated with 5 µM 5(6)-carboxy-2'.7'-dichloro-fluorescein diacetate (CDFDA) for 30 min at 37 °C and then analyzed on the fluorescence microscope Axio Observer.D1 (objective EC Plan-Neofluar 5x/0.16 Ph 1) under transmitted light and after excitation with 470 nm at 525 nm. The membrane-bound non-fluorescent CDFDA is intracellularly converted by esterases into the green fluorescent ABCC2 substrate 5(6)-carboxy-2'.7'-dichlorofluorescein (CDF). By ABCC2-mediated transport, CDF enters the bile ducts. Representative sections are shown. The indicated scale applies to all images.

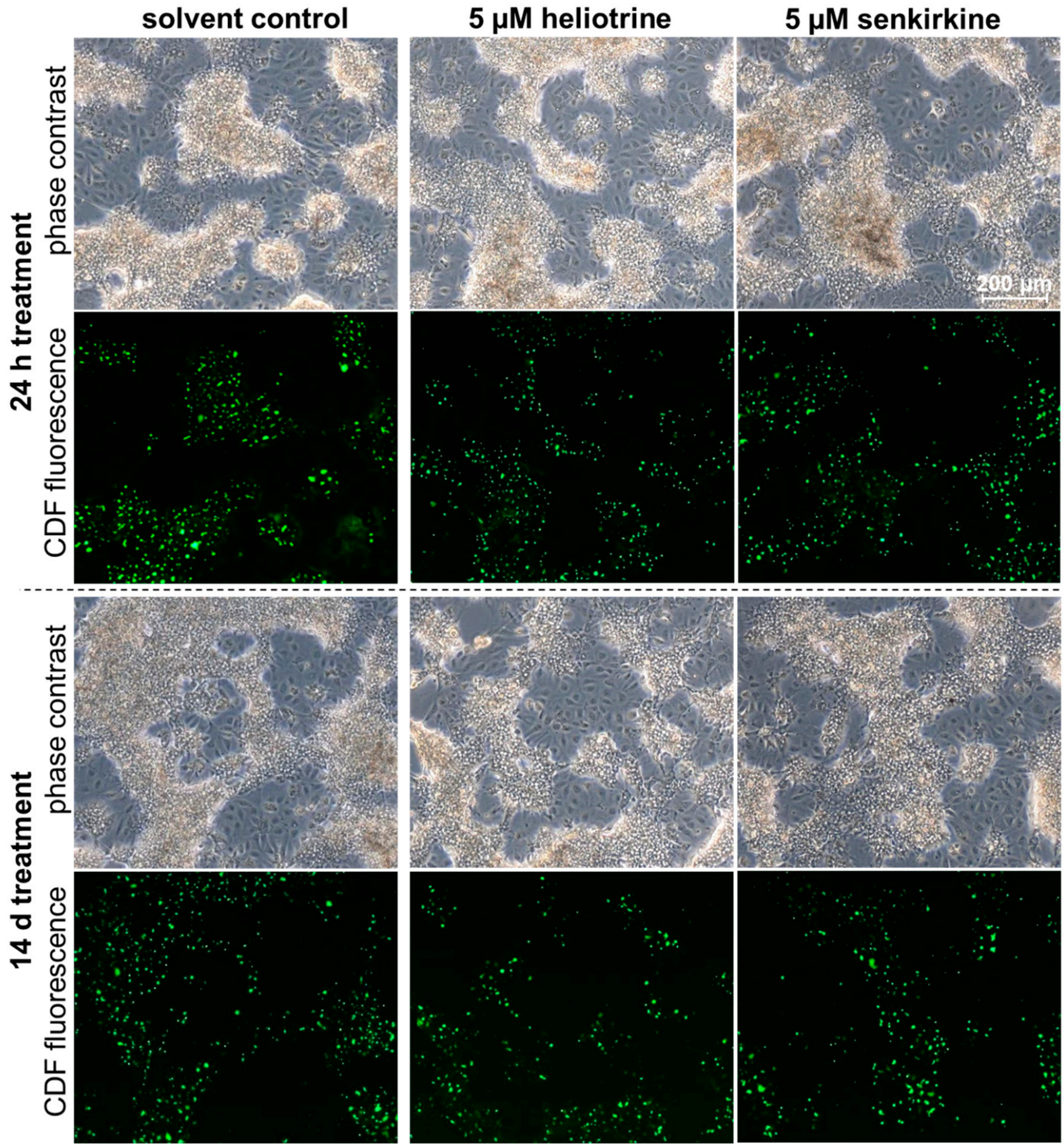

Figure S3: PA-dependent disturbance of ABCC-2 driven efflux in HepaRG cells: Differentiated HepaRG cells were incubated for 24 h or 14 days with 5 µM heliotrine or senkirkine as well as with the solvent (1.7 % DMSO and 0.35 % ACN). To localize the bile canaliculi, the cells were incubated with 5 µM 5(6)-carboxy-2'.7'-dichloro-fluorescein diacetate (CDFDA) for 30 min at 37 °C and then analyzed on the fluorescence microscope Axio Observer.D1 (objective EC Plan-Neofluar 5x/0.16 Ph 1) under transmitted light and after excitation with 470 nm at 525 nm. The membrane-bound non-fluorescent CDFDA is intracellularly converted by esterases into the green fluorescent ABCC2 substrate 5(6)-carboxy-2'.7'-dichlorofluorescein (CDF). By ABCC2-mediated transport, CDF enters the bile ducts. Representative sections are shown. The indicated scale applies to all images.

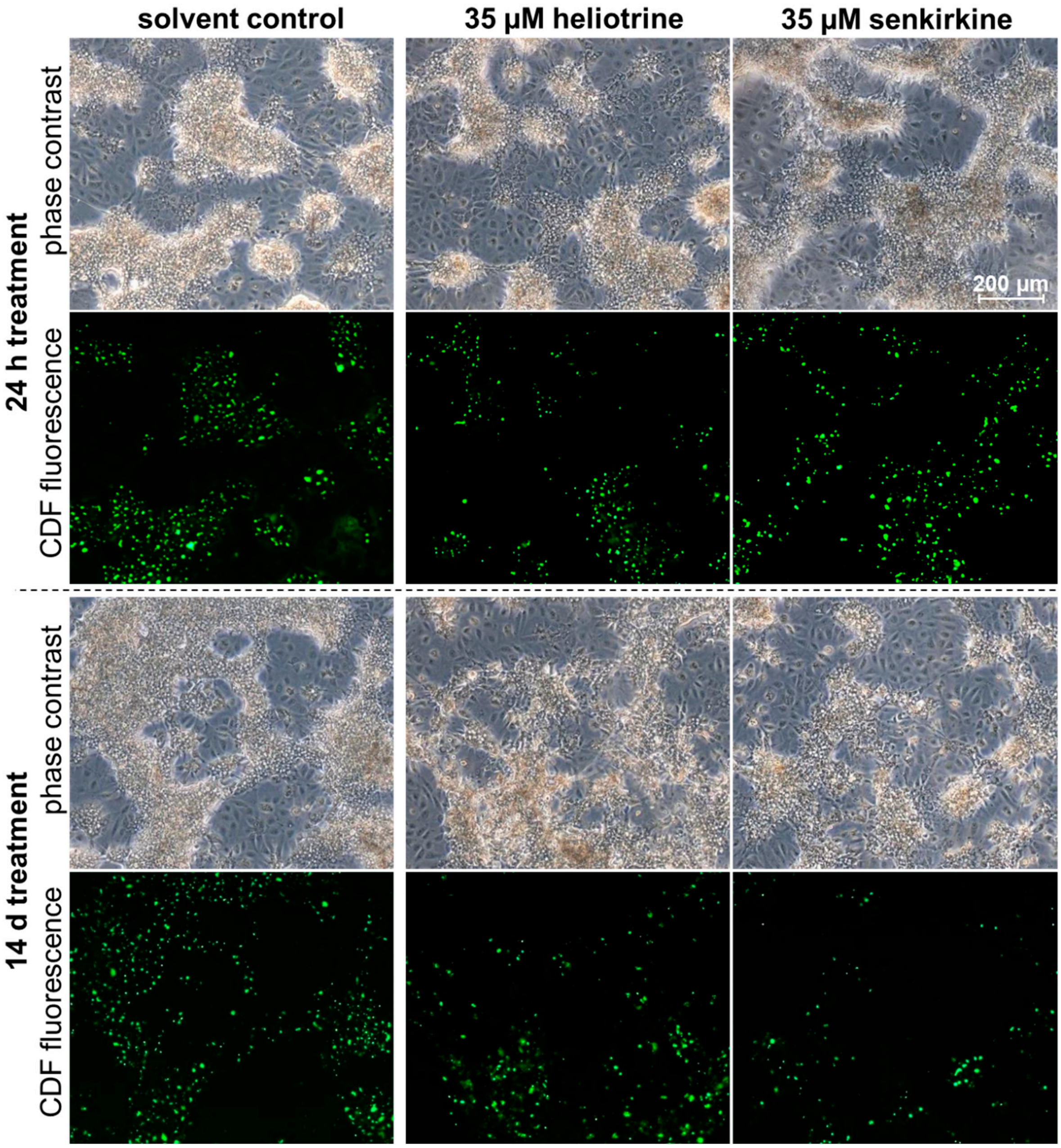

Figure S4: PA-dependent disturbance of ABCC-2 driven efflux in HepaRG cells: Differentiated HepaRG cells were incubated for 24 h or 14 days with 35 µM heliotrine or senkirkine as well as with the solvent (1.7 % DMSO and 0.35 % ACN). To localize the bile canaliculi, the cells were incubated with 5 µM 5(6)-carboxy-2'.7'-dichloro-fluorescein diacetate (CDFDA) for 30 min at 37 °C and then analyzed on the fluorescence microscope Axio Observer.D1 (objective EC Plan-Neofluar 5x/0.16 Ph 1) under transmitted light and after excitation with 470 nm at 525 nm. The membrane-bound non-fluorescent CDFDA is intracellularly converted by esterases into the green fluorescent ABCC2 substrate 5(6)-carboxy-2'.7'-dichlorofluorescein (CDF). By ABCC2-mediated transport, CDF enters the bile ducts. Representative sections are shown. The indicated scale applies to all images.
